# Identification and Structural Characterization of Degradation Products of Linagliptin by Mass Spectrometry Techniques

**DOI:** 10.3390/ijms25052591

**Published:** 2024-02-23

**Authors:** Aleksandra Liana, Adam Hałuszczuk, Andrzej Gawor, Ewa Bulska

**Affiliations:** 1 Biological and Chemical Research Centre, Faculty of Chemistry, University of Warsaw, Żwirki i Wigury 101, 02-089 Warsaw, Poland; aleksandra.liana@polpharma.com (A.L.); agawor@chem.uw.edu.pl (A.G.); 2 Pharmaceutical Plant Polpharma S.A., Pelpińska 19, 83-200 Starograd Gdański, Poland; 3 Faculty of Chemistry, Gdańsk University of Technology, Gabriela Narutowicza 11/12, 80-233 Gdańsk, Poland; ahaluszczuk@gmail.com

**Keywords:** degradation, degradation profile, linagliptin, impurity identification, LC-PDA-MS, LC-Q-ToF-MS

## Abstract

As part of the development and production of pharmaceuticals, the purity of Active Pharmaceutical Ingredients stands as a fundamental parameter that significantly influences the quality, safety, and efficacy of the final drug product. Impurities in Active Pharmaceutical Ingredients are various unwanted substances that can appear during the whole manufacturing process, from raw materials to the final product. These impurities can stem from multiple sources, including starting materials, intermediates, reagents, solvents, and even degradation products resulting from exposure to environmental factors such as heat, light, or moisture. Their presence can potentially compromise the therapeutic effect of the drug, introduce unexpected side effects, or even pose safety risks to patients. This study aims to conduct the forced degradation of linagliptin and subsequently attempt to identify the resulting degradants. The degradation procedures were carried out in accordance with the guidelines of the International Committee for Harmonization. The degradation profile of linagliptin was investigated under various conditions, including acid hydrolysis, alkaline hydrolysis, oxidation, heat, and light exposure, utilizing ultra-performance liquid chromatography connected to a photo array detector. Identification and characterization of the degradation products were achieved using an ultra-performance liquid chromatography coupled with a single quadrupole detector mass spectrometer and also a liquid chromatography coupled with a high-resolution mass spectrometry. The identified degradation products demonstrate that linagliptin is particularly susceptible to degradation when exposed to acid and peroxide. Whereas, no significant degradation effects were observed under alkali, thermolytic, and photolytic conditions.

## 1. Introduction

In the pharmaceutical industry, a highly significant parameter characterizing Active Pharmaceutical Ingredients (APIs) is their chemical purity [1]. The presence of diverse chemical impurities can result in a reduction in the efficacy of the API’s by delivering a lower amount of the active substance to the patient. Furthermore, these impurities might potentially exhibit toxic properties, posing a notable risk to patient safety. The term “impurities” refers to all undesirable substances that may be present in the final product resulting from a given API synthesis [2,3,4]. In practice, no chemical reaction exhibits absolute selectivity, and no chemical compound remains entirely stable. Hence, the knowledge of their chemical structure and a comprehensive understanding of the mechanisms of their formation is crucial to improving the synthetic process and mitigating, or even eliminating these impurities from drug substances.

In accordance with the definition provided by the United States Pharmacopeia (USP) [5], an impurity is “any component of a drug substance that is not the chemical entity defined as the drug substance and in addition, for a drug product, any component that is not a formulation ingredient”. Considering that impurities can originate from various sources, its proper classification is essential. According to the International Council for Harmonization (ICH) [6], impurities can be categorized as organic, inorganic, and residual solvents.

Organic impurities may include starting materials, byproducts, intermediates, reagents, ligands, and catalysts, as well as degradation products. Inorganic impurities include reagents, ligands, catalysts, heavy metals, other metal residues, inorganic salts, and additional materials such as filtration aids, silica, and activated charcoal. The last group comprises solvents used in synthesis, which can be both organic and inorganic liquids. This category holds a recognized level of toxicity, and the requirements concerning their presence in the active substance are outlined in the ICH Q3 series guidelines [6]. One of the mentioned impurities groups outlined in the guidelines is degradation products. ICH guidelines are mandatory for API manufacturers and obligate them to provide stability degradation studies under a range of different conditions, such as acidic and basic hydrolysis, light irradiation, oxidation, high-temperature dry heat, and increased humidity [7]. Various combinations of liquid chromatography (LC) and mass spectrometry (MS) techniques [4,8,9,10,11] are utilized to identify degradation products in Active Pharmaceutical Ingredients. A notable approach involves using ultra-performance liquid chromatography coupled with a single quadrupole detector mass spectrometer (LC-PDA-MS).

This study aims to perform the forced degradation of linagliptin and attempt to identify the main degradants formed under the influence of various stress factors. Linagliptin [12] is an active pharmaceutical compound belonging to a class of medications known as dipeptidyl peptidase-4 (DPP-4) inhibitors. It is utilized by patients with type 2 diabetes mellitus (T2DM), a condition characterized by elevated blood sugar levels due to the body’s impaired production or utilization of insulin. Administered orally, linagliptin functions by augmenting the levels of specific natural substances that effectively lower elevated blood sugar levels [13]. Linagliptin is chemically designated as 8-[(3R)-3-aminopiperidin-1-yl]-7-(but-2-yn-1-yl)-3-methyl-1-[(4-methylquinazolin-2yl)methyl]-3,7-dihydro-1H-purine-2,6-dione. The empirical formula is C_25_H_28_N_8_O_2_ and the molecular weight is 472.54 g/mol [14]. In the available literature, we can only find information about some structures of the resulting degradants [15,16,17]. There is a lack of information on a comprehensive analysis of degradation products, as well as proposed structures for the identified impurities. Several studies have been conducted on the assessment of linagliptin through liquid chromatography methods, encompassing both HPLC and UPLC, in conjunction with UV or PDA detectors [17,18,19,20,21,22,23,24]. However, there are not many papers related to the identification of impurities. The available literature presents the identification of process impurities [15,25,26,27,28]. It is essential to note that these impurities may vary among different producers, due to the distinct synthesis routes for linagliptin. Additionally, only a few publications related to linagliptin degradation were identified [16,22]. Unfortunately, these studies do not comprehensively cover the topic, and there is still insufficient data on the formation of all degradation impurities resulting from exposure to temperature, light, as well as oxidizing environments or acid and alkaline hydrolysis. The proposed forced degradation study provides valuable insights into the test substance. It not only aids in recognizing the chemical properties of drug molecules but also highlights the substance’s sensitivity under different conditions. Furthermore, such studies can assist in resolving stability-related issues and unveil the degradation mechanisms of the drug substance. This knowledge is instrumental in refining the synthesis process by avoiding critical conditions that lead to the formation of undesirable degradants. The novelty introduced in this study lies in the approach employed during forced degradation investigations. It involves subjecting substances to stringent conditions, enabling the generation of the full spectrum of potential impurities. Particular attention was paid to validate whether the applied analytical method can reliably identify these impurities during routine testing. Forced degradation processes often yield impurities at higher levels compared to those formed during synthesis or natural degradation. A comprehensive understanding of the chemical structure of these impurities holds the key to assessing their potential toxicological impacts. Moreover, having insights into the reaction mechanisms and pinpointing the stage at which a particular impurity is formed is of paramount importance for ensuring the consistent, high-quality production of pharmaceutical substances.

As a result of forced degradation, a degradation profile is produced, similar to that of what would be observed in a formal stability study under ICH conditions. In addition, based on these data, it was possible to select a more appropriate purification method for the tested compound. The purpose of this research was to trace the degradation of linagliptin under various stress conditions, i.e., acidic and basic hydrolysis, oxidation, dry heat, and light degradation, and propose the structure of some of the resulting degradants.

## 2. Results

**Figure 1:**
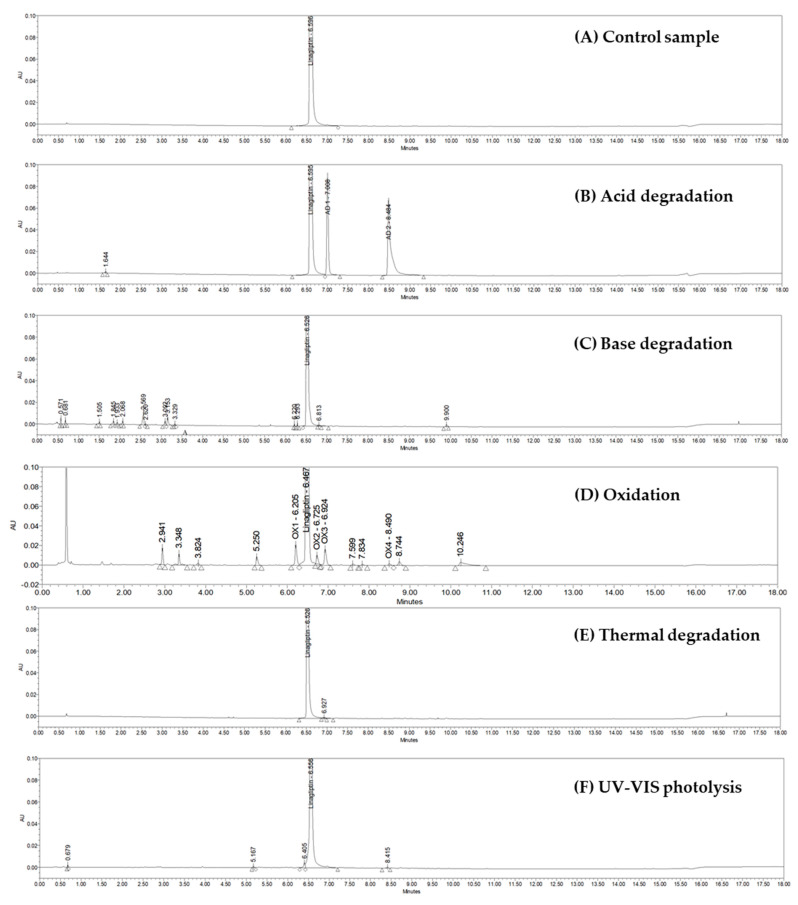
Forced degradation study chromatograms: (**A**) control sample (pure linagliptin); (**B**) exposed to acid degradation; (**C**) exposed to base degradation; (**D**) exposed to oxidation (H_2_O_2_); (**E**) exposed to thermal degradation, and (**F**) exposed to UV-VIS photolysis.

**Figure 2:**
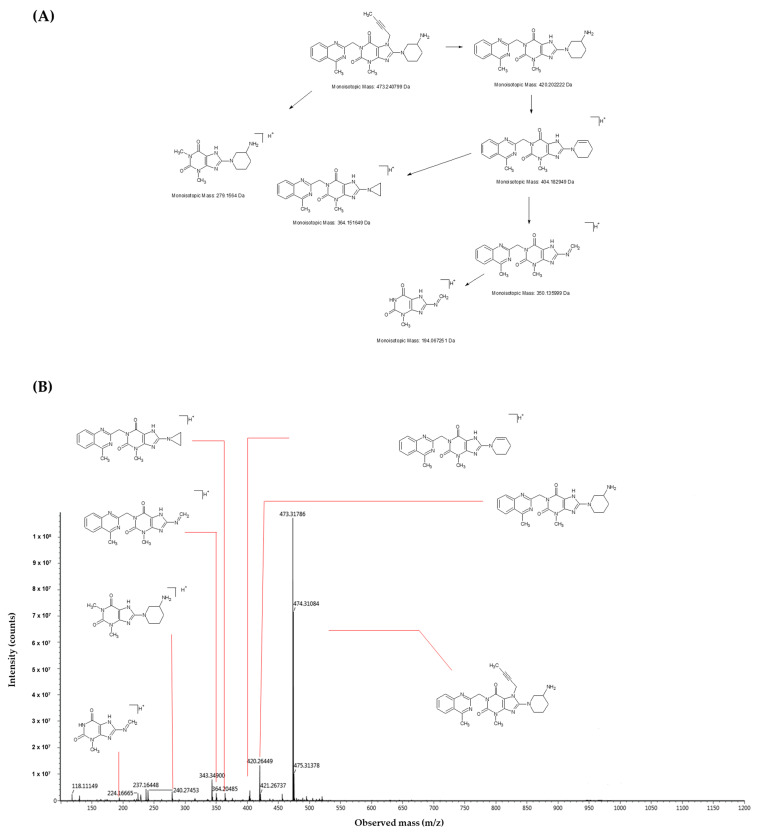
Electrospray positive ionization—mass spectrometry (ESI-MS) spectrum of linagliptin recorded using the XEVO G2-XS Q-ToF high resolution mass spectrometer (**B**) and proposed fragmentation pattern of Linagliptin (**A**).

Ensuring the stability and efficacy of pharmaceutical compounds is of utmost importance in healthcare. In line with this, the degradation behavior of linagliptin, a significant dipeptidyl peptidase-4 (DPP-4) inhibitor, has been extensively investigated. The study focuses on its response to diverse conditions, including acid and alkaline hydrolysis, oxidation, heat, and light exposure. Additionally, the selection of specific conditions was based on the most commonly recognized harmful conditions for the drug production process, and the temperature was adjusted to obtain the highest number of degradants in a short period, based on ICH recommendations. Samples, taken at intervals, were analyzed using an ultra-performance liquid chromatography with a photo array detector. This exploration offers insights into linagliptin’s stability and guides strategies for maintaining its quality over time. In the following sections, we will explore the methodologies employed and the key findings that shed light on the compound’s behavior under various stressors. It was assumed that as a result of forced degradation, an obtained degradation profile was similar to that of what would be observed using a formal stability study under ICH conditions. The resulting degradants were determined using an ultra-performance liquid chromatography (UHPLC) combined with a photodiode array detector. The analytical method used was validated based on the ICH guideline Q2(R2)—“Validation of Analytical Procedures” [29]. Identification and characterization of the degradation products were achieved using an ultra-performance liquid chromatography coupled with a single quadrupole detector mass spectrometer (LC-SQD), and also using liquid chromatography coupled with a high-resolution mass spectrometry (LC-Q-ToF-MS). The results are presented in Figure 1 and Figure 2, and Table 1. Initial analysis involved meticulous examination of the mass spectra of linagliptin, which served as a foundation for impurity identification. By comparing characteristic fragments obtained from linagliptin with those from degraded samples, appropriate structures for the observed impurities were proposed. Moreover, we carefully considered the specific conditions under which these impurities were formed, offering insight into the potential mechanisms driving their generation.

### 2.1. Results of the Linagliptin without and after Forced Degradation

#### 2.1.1. Linagliptin Solution without Degradation

The sample of linagliptin that was analyzed had not undergone any degradation. The retention time of linagliptin in the chromatogram was 6.60 min. The analysis revealed that the linagliptin sample used for the research was 100% pure, with all impurities falling below the detection limit of the method (0.02%).

#### 2.1.2. Acidic Degradation

It was found that 16.42% degradation occurred when the linagliptin solution was subjected to acid degradation for 24 h at 60 °C. Notably, substantial degradation was observed during the acid hydrolysis of linagliptin. The impurities with retention times of 7.008 min (relative retention time: 1.06 min, identified as AD 1) and 8.484 min (relative retention time: 1.29 min, identified as AD 2) were formed at levels exceeding 5.0%.

#### 2.1.3. Basic Degradation

It was found that 2.56% degradation occurred when the linagliptin solution was subjected to alkaline degradation for a duration of 10 days at 60 °C. Base hydrolysis did not cause significant degradation of linagliptin. However, after the 10-day degradation period, two degradants exceeding 0.4% were observed: one at a retention time of 2.569 min (with a relative retention time of 0.39 min) and another at a retention time of 3.153 min (with a relative retention time of 0.483 min). Additionally, several impurities emerged, both below 0.1%.

#### 2.1.4. Oxidative Degradation

It was found that 35.86% degradation occurred when the linagliptin solution underwent oxidative degradation for 24 h at 60 °C. Significant degradation of linagliptin was observed under oxidative conditions. Consequently, several impurities above 0.4% were generated: one at a retention time of 2.941 min (with a relative retention time of 0.46 min), another at 3.348 min (with a relative retention time of 0.52 min), followed by 5.250 min (0.81 min), 6.205 min (0.96 min, identified as OX 1), 6.725 min (1.04 min, identified as OX 2), 7.924 min (1.07 min, identified as OX 3), 8.490 min (1.31 min, identified as OX 4), 8.744 min (1.35 min), 10.246 (1.58) and additional impurities formed at levels below 0.1%.

#### 2.1.5. Thermal Degradation

It was found that 0.05% degradation occurred when the linagliptin substance was subjected to thermal degradation for a period of 10 days at 60 °C. No significant degradation of linagliptin was observed during the thermal decomposition process. One impurity was formed at a level of 0.05%.

#### 2.1.6. UV-VIS Photolysis

It was found that 0.56% degradation occurred when the linagliptin substance was subjected to UV-VIS degradation at 60 °C and 60% humidity. No significant degradation of linagliptin was observed during the UV-VIS photolysis. Several impurities emerged, both below 0.1%.

A summary of the obtained results is presented in Table 2. These findings provide insight into the stability of linagliptin under different degradation conditions, highlighting the impact of acidic and oxidative environments on its degradation. The temperature of 60 degrees Celsius was chosen for the degradation study of linagliptin, due to its common occurrence in the literature [30] as a representation of extreme conditions to which it may be exposed due to improper storage or heating of the preparation. Additionally, this temperature is sufficiently high to generate impurities visible for MS, allowing for a thorough examination of the degradation profiles.

### 2.2. Validation Parameters

The analytical procedure employed for assessing the purity of samples before and after degradation has been duly validated following the guidelines established by the ICH, specifically as outlined in ICH Q2(R2)—“Validation of Analytical Procedures” [29]. In order to carry out the validation of the liquid chromatography method designed for purity testing of linagliptin substance, the following parameters of the method were checked: specificity, linearity, accuracy, limit of quantitation, limit of detection, precision, range, stability, and robustness.

#### 2.2.1. Specificity

No discernible interference was detected among the main peak, diluent peaks, and impurity peaks. The resolution factors (R_s_) between the peaks originating from specified impurities and linagliptin consistently exceeded the threshold of 1.5.

#### 2.2.2. Linearity

The linearity of the responses for both the linagliptin standard substance and the known impurities was evaluated across a concentration range from 0.05% to 0.25% (concerning the linagliptin concentration in the test solution, set at 1.0 mg/mL). The acceptance criteria were met, with the correlation coefficient (r) of the regression curve for all substances not falling below 0.99, and the residuals remaining within the range of ±20.0%.

#### 2.2.3. Accuracy

The method’s accuracy was substantiated through recovery calculations. In an assessment of accuracy, test solutions of linagliptin test substance were fortified with known impurities, covering a concentration range from 0.05% to 0.20% (concerning the linagliptin concentration in the test solution, maintained at 1 mg/mL). The acceptance criterion was fulfilled as the recovery values fell within the range of 80.0% to 120.0%.

#### 2.2.4. Limit of Detection and Limit of Quantitation

The limit of detection (LOD) and limit of quantitation (LOQ) were estimated based on the calculated data of signal-to-noise ratio (S/N) from the linearity testing. For the LOQ, a content level of 0.05% was established for both linagliptin and the identified impurities (concerning the linagliptin concentration in the test solution—1 mg/mL). This decision was predicated on the S/N ratio exceeding 10, accompanied by the method’s demonstrated linearity, accuracy, and precision at this threshold. Conversely, for specified impurities, a content level of 0.01% for both linagliptin and identified impurities (relative to the concentration of linagliptin in the test solution—1 mg/mL) was established as LOD. This determination was substantiated by S/N ratios surpassing 3 at these levels.

#### 2.2.5. Precision

To assess method precision, six injections of linagliptin and impurities solution at a 0.15% concentration (relative to the linagliptin concentration in the test solution of 1 mg/mL) were performed. Subsequently, the relative standard deviation (RSD) for peak areas was calculated. The acceptance criteria, which stipulate that the RSD for peak areas of both linagliptin and impurities at the 0.15% level should not exceed 10.0%, was duly satisfied, demonstrating the method’s precision.

For the evaluation of repeatability, six test solutions of linagliptin were prepared and spiked with known impurities at the 0.15% level. This test was conducted by a single analyst over the course of a single day. The acceptance criteria, comprising recovery values of impurities falling within the range of 80.0% to 120.0% and an RSD of recovery results not exceeding 10.0% (n = 6), were satisfactorily met.

To gauge reproducibility, an identical set of six test solutions of linagliptin, enriched with known impurities at the 0.15% level, were prepared. The test was conducted on a different day, by a different analyst, and in a distinct laboratory, as compared to the repeatability analysis. Moreover, a different ultra-high-performance chromatograph and chromatography column were employed than those used in the repeatability analysis. The established acceptance criteria for recovery values of impurities and RSD of recovery results, both individually and collectively for combined data from the repeatability and reproducibility tests (n = 12), were rigorously met.

#### 2.2.6. Measurement Range

Based on the results of the linearity, accuracy, and precision tests, it can be concluded that this method is suitable for the analysis of both known and unspecified impurities in the linagliptin substance within the concentration range of 0.05% to 0.20% (relative to the linagliptin concentration in the test solution—1 mg/mL).

#### 2.2.7. Stability of the Test Substance

To assess the stability of the solutions, test solutions fortified with known impurities and the test solution were prepared. These solutions were analyzed immediately after preparation and subsequently at intervals of 6, 12, 24, 48, and 72 h. Storage conditions included room temperature (25 °C) and autosampler temperature (10 °C). The acceptance criterion established that the difference between peak areas measured immediately after preparation and at specified time intervals should not exceed ±10.0%. The study findings indicate that both the test solution spiked with known impurities and the test solution can be safely stored for up to 24 h after preparation at a temperature of 25 °C and for up to 72 h at a temperature of 10 °C.

#### 2.2.8. Robustness

The method is robust for changing the column temperature by 45 °C ± 5 °C, the ammonia concentration in mobile phase B (0.1% NH_3_ in H_2_O/MeOH 9/1) by 0.1% ± 0.01%, and the mobile phase B (acetonitrile) in gradient by 3% ± 1%.

## 3. Discussion

The results of this study underscore the significance of monitoring the stability of pharmaceutical compounds to ensure their safety and efficacy. Linagliptin is a highly chemically active compound with a complex molecular structure. This chemical reactivity is a consequence of its unique structure, which includes various functional groups and bonds that can readily interact with other substances. The chemical activity of linagliptin underpins its utility in pharmaceutical applications, allowing for the synthesis of derivatives, the formation of complexes, and engagement in intricate biochemical processes. This characteristic also underscores the need for careful handling and storage to prevent undesired chemical transformations that could affect its stability and efficacy. The understanding of linagliptin chemical reactivity is crucial for optimizing its formulation, ensuring its therapeutic effectiveness, and minimizing the potential for unintended reactions. This aligns with the existing literature, highlighting the importance of degradation studies in identifying potential impurities that could impact drug quality and patient health. The forced degradation study showcases the compound’s sensitivity to acid and peroxide stress conditions. This observation resonates with earlier research conducted by Attimarad et al. [18], that emphasized the susceptibility of linagliptin tablets to forced degradation. The emergence of degradants emphasizes the necessity of monitoring even minor impurities that could potentially impact product quality. The identification of specific impurities provides valuable insights into the acid and oxidation degradation mechanisms. Concerning the obtained MS data, and drawing upon the available literature, we endeavor to elucidate plausible mechanisms underlying the formation of acid-induced and oxidative degradation products of linagliptin. The proposed degradation products were authenticated through commercially available standards and by synthesizing and comparing individual compounds with high-resolution MS spectra. In the ‘Supplementary Material’ section (Figures S1–S5), the MS spectra and fragmentation pathways for all analyzed impurities are provided.

**Figure 3:**
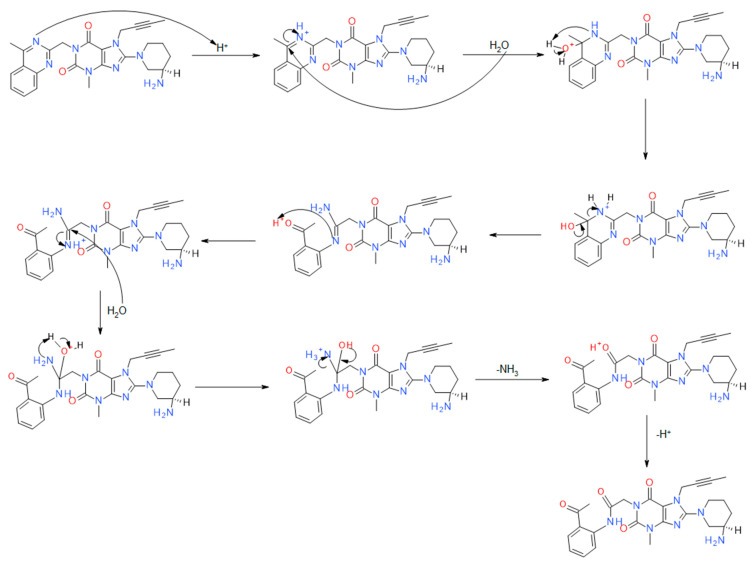
Proposed reaction mechanism of the impurity resulting from acid degradation—AD1.

**Figure 4:**
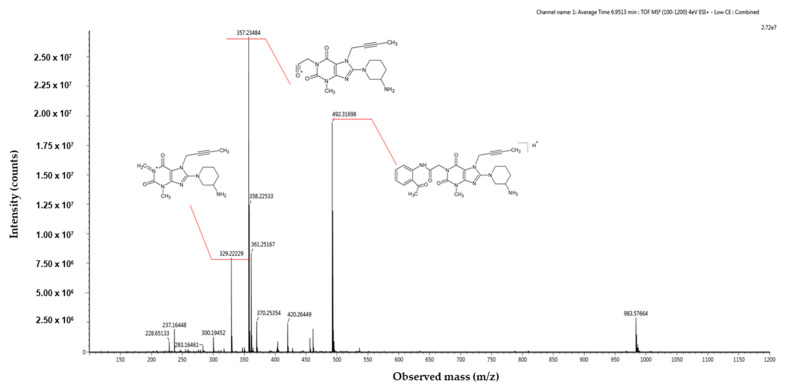
High-resolution mass spectrometry spectrum of AD1 impurity resulting from acid degradation in positive ionization mode.

With regard to acidic degradation, we propose the following mechanistic pathways. The AD1 impurity arises from the partial hydrolysis of the quinazoline ring (Figure 3). Hydrolysis under acidic conditions is a characteristic feature of this heterocyclic system. To confirm the suggested molecular structures from the proposed reaction mechanism, measurements were carried out using a high-resolution mass spectrometer. Figure 4 illustrates the acquired mass spectra, accompanied by the structural representations of the identified compounds, which are the predominant fragmentation products detected at mass-to-charge ratios of 492.31698 *m*/*z*, 357.23484 *m*/*z*, and 329.22229 *m*/*z*. Importantly, the impurities were denoted as AD1, which we verified in our investigation. This finding is consistent with previous studies conducted by Yadav et al. [16] and Huang et al. [15], who reported similar impurities under comparable conditions. The retention time consistency observed in chromatograms further supports the reliability of our identification.

**Figure 5:**
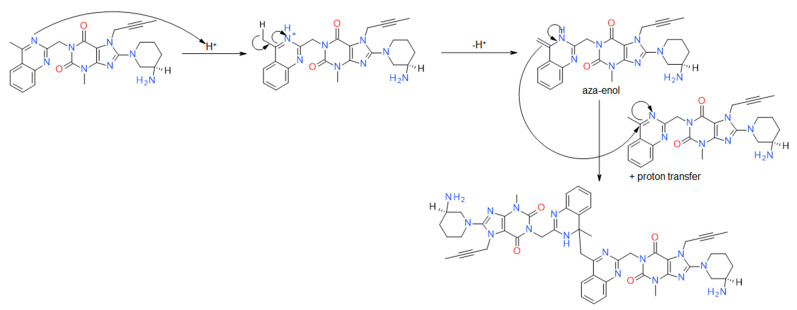
Proposed reaction mechanism of the impurity resulting from acid degradation—AD2.

The AD2 impurity is linagliptin, which has dimerized through acid-catalyzed aza-enolization (Figure 5). The resulting Schiff base subsequently initiates a nucleophilic attack on the quinoline ring of another linagliptin molecule acting as an electrophile.

**Figure 6:**
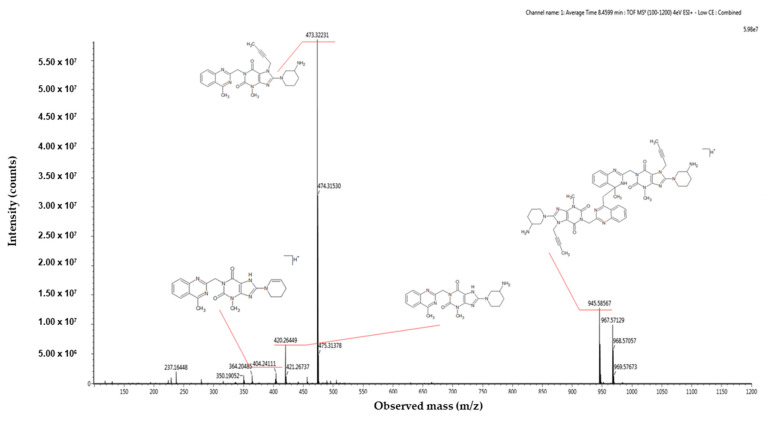
High-resolution mass spectrometry spectrum of AD2 impurity resulting from acid degradation in positive ionization mode.

To validate its structure, we synthesized the impurity and subjected it to comprehensive characterization using MS and NMR techniques. Structural confirmation not only affirmed our identification, but also provided valuable insights into the potential mechanisms underlying its formation. As presented in Figure 6, the obtained mass spectra are presented alongside the structural depictions of the identified compounds. These compounds represent the fragmentation products detected at specific mass-to-charge ratios: 945.58567 *m*/*z*, 473.32231 *m*/*z*, 420.26449 *m*/*z*, 404.24111 *m*/*z*, 364.20485 *m*/*z*, 350.19052 *m*/*z*, and 237.164848 *m*/*z*. The impurities were synthetized by an external investigation and were confirmed through MS and NMR spectra. The comprehensive literature research revealed that there are no studies related to the identification this impurity.

In the case of impurities resulting from oxidative degradation, our identification process relied primarily on MS spectra, complemented by chemical knowledge and the conditions of impurity formation. While comparison with the existing literature was conducted, structural confirmation through alternative techniques was not pursued. In order to confirm the predicted molecular structures based on the proposed reaction mechanism, we conducted measurements using a high-resolution mass spectrometer.

The results of the experiment support the OX 1 impurity resulting from the oxidation of the tertiary nitrogen in the pyridinium ring. This principle extends to amines containing aromatic substituents, as they are typically more susceptible to oxidation. Conversely, amines located within aromatic rings, such as the pyridine type, tend to possess a higher oxidation potential and are less prone to oxidation. Hence, we propose that the mass [M + H]^+^ = 489.32132 corresponds to the formation of piperidine N-oxide rather than quinazoline oxidation.

Further oxidative degradation products were probably also obtained by Yadav et al. [16]. They proposed the structure of degradants giving [M + H]^+^ ions at *m*/*z* 487 as linagliptin oxidized both on the tertiary (yielding N-oxide) and the primary nitrogen (yielding imine). They described these impurities as two isomers of such compounds. However, the presence of an imine with sp2 electron configuration in the piperidinium ring would preclude geometric isomerism in such a case; therefore, in our opinion, it is more plausible that these degradants constitute the Z and E isomers of the oxime formed as a result of primary nitrogen oxidation (OX 2 and OX 3). The OX 4 impurity is a linagliptin derivative in which the primary amine was converted to its corresponding nitro compound. In the case of impurities resulting from oxidative degradation, our identification process relied primarily on the MS spectra, complemented by chemical knowledge and the conditions of impurity formation. While a comparison with the existing literature was conducted, structural confirmation through alternative techniques was not pursued.

Forced degradation testing plays a pivotal role in pharmaceutical research and development, serving multiple crucial purposes. Primarily, it facilitates the establishment of degradation pathways for drug substances and products, elucidating mechanisms such as hydrolysis, oxidation, thermolysis, or photolysis. By inducing impurities under diverse conditions, a degradation profile similar to that observed in formal stability studies under ICH conditions can be obtained. Furthermore, forced degradation testing aids in resolving stability-related issues, by providing insights into the compound’s susceptibility to various stressors. In the case of linagliptin, forced degradation testing enabled the evaluation of the suitability of our developed chromatographic method for routine testing during production and stability assessments. One of the goals of forced degradation is to obtain an impurity profile similar to that obtained from official stability tests. Notably, it was observed that some impurities may form during synthesis, emphasizing the importance of optimizing reaction conditions to minimize their occurrence. Our tests highlighted linagliptin’s vulnerability to acidic and oxidizing conditions, necessitating the avoidance of strongly acidic environments during synthesis and the use of appropriate antioxidants to mitigate specific impurities. During forced degradation tests, it was noticed that it was not sensitive to temperature and humidity. Thanks to this, there is no need to apply special storage conditions. However, formal stability studies conducted according to ICH guidelines [6,7,29] determine storage conditions and stability durations. Forced degradation testing provides invaluable insights into the stability profile of linagliptin, guiding the optimization of synthesis conditions, the selection of antioxidants, and the development of storage protocols. By addressing the stability-related challenges early in the development process, the production of high-quality linagliptin formulations with enhanced stability and efficacy can be ensured.

In the rapidly advancing field of analytical chemistry, the integration of machine learning techniques holds significant promise for enhancing our analytical capabilities. Recent papers [31,32,33] underscore the notable progress made in this area and highlight the potential for augmenting our analytical methodologies. Although our current study focuses on traditional analytical approaches, such as chromatography and mass spectrometry, the integration of machine learning algorithms can offer a myriad of benefits. These benefits include improved efficiency and accuracy in impurity identification, enhanced data interpretation, and overall analytical performance optimization. Furthermore, further automation of the protocols proposed in our study could contribute to increased efficiency and reproducibility in pharmaceutical research and development endeavors.

## 4. Materials and Methods

### 4.1. Chemical and Reagents

The linagliptin was obtained from Pharmaceutical Plant Polpharma S.A. (Starogard Gdański, Poland). Acetonitrile (HPLC grade) was purchased from J.T. Backer (Phillipsburg, NJ, USA). Hydrochloric acid (0.1 mol/L), sodium hydroxide (0.1 mol/L), 25% ammonia solution (for LC-MS), and 30% hydrogen peroxide solution were purchased from Merck (Darmstadt, Germany). Ultra-pure water was obtained from the MilliQ water system (Millipore Integral Water Purification System, Darmstadt, Germany).

### 4.2. Instrumentation

Thermal, acid, base, and oxidative degradation were performed at elevated temperatures using a dryer-type ED 53 (Binder, Tuttlingen, Germany). The dryer is equipped with a digital temperature mechanism enabling temperature control. Photodegradation was carried out in a climatic chamber for photostability studies, type KBF 240 LQC E6, equipped with a UV-VIS lamp, capable of controlling the temperature and humidity (Binder, Tuttlingen, Germany). The forced degradation samples were analyzed on a UPLC Aquality system equipped with a binary pump, autosampler, column compartment, and photodiode array detector PDA (Waters, Eschborn, Germany), controlled by Empower software (version no. 7.30.00.00). Identification of degradants was performed using UPLC Aquality system equipped with a binary pump, autosampler, column compartment, and a photodiode array detector PDA connected with a single quadrupole detector (SQD) mass spectrometer (Waters, Eschborn, Germany), controlled by Empower software (version no. 7.30.00.00). To confirm the structures of degradation compounds, an ultra-performance liquid chromatography system, Acquity I-Class BSM Plus, equipped with a binary pump, autosampler, and column compartment, was used in conjunction with a photodiode array detector (UPLC-PDA) and a high-resolution mass spectrometer XEVO G2-XS with Q-ToF mass analyzers (Waters, Eschborn, Germany), controlled by Unifi software (UNIFI^®^ Scientific Information System, version no. 1.8) (Waters, Eschborn, Germany).

### 4.3. Sample Preparation

The procedure for preparing solutions used in forced degradation tests is presented below. The description includes detailed information on the preparation of the diluent, the linagliptin sample used in the study, and the linagliptin samples exposed to various stress factors:

- Diluent solution for sample preparation

The solution used to dissolve the samples in sample preparation for analysis contained acetonitrile and water in a 1:1 volume ratio. The solution was tested.

- Linagliptin solution without degradation

A total of 52.25 mg of the linagliptin was accurately weighed into a 50 mL volumetric flask, dissolved in a diluent, filled to the volume with a diluent, and mixed. The solution was tested.

- Acidic hydrolysis

A total of 52.36 mg of linagliptin was accurately weighed into a 50 mL volumetric flask, mixed with 5 mL of 0.1 mol/L HCl solution, and placed in an oven at 60 °C. After 24 h, the solution was cooled down and 5 mL of 0.1 mol/L NaOH was added to neutralize the solution. Next, 15 mL of water was added, filled up to volume with acetonitrile, and mixed. The solution was tested.

- Basic hydrolysis

A total of 50.03 mg of linagliptin was accurately weighed into a 50 mL volumetric flask, mixed with 5 mL of 0.1 mol/L NaOH solution, and placed in an oven at 60 °C. After 10 days, the suspension was cooled down, and 5 mL of 0.1 mol/L HCl was added to neutralize the suspension. Next, 15 mL of water was added, filled up to volume with acetonitrile, and mixed. The solution was tested.

- Oxidation

A total of 50.62 mg of the linagliptin was accurately weighed into a 50 mL volumetric flask and mixed with 5 mL of 3% H_2_O_2_ solution and placed in an oven at 60 °C. After 24 h, the suspension was cooled down, and 20 mL of water was added. Next, acetonitrile was filled up to into the volume. The solution was tested.

- Thermal degradation

About 1.0 g of linagliptin was placed in an oven at 60 °C for 10 days. Next, 50.23 mg of degradation product substance was weighed into 50 mL volumetric flask, dissolved in a diluent, filled to the volume with a diluent, and mixed. The solution was tested.

- UV-VIS photolysis

The linagliptin was exposed to visible light (2.4 million lux hours), and near-ultraviolet light (400-watt hours/square meter). Also, a placed and protected from the light was another sample of Linagliptin test substance as a dark control in the same chamber. Working conditions of the chamber were: temperature 60 °C and relative humidity 60%. Next, 50.00 mg of test substance after UV-VIS exposure and dark control sample were weighed into a 50 mL volumetric flask, dissolved in a diluent, filled up to the volume with diluent, and mixed. The solutions were tested.

### 4.4. UPLC-PDA Condition

The chromatographic separation was performed on UPLC BEH C18 (2.1 mm × 100 mm; 1.7 µm) column (Waters, Eschborn, Germany), using reverse phase gradient elution. The gradient method involves two mobile phases (A and B). The composite of these solutions in the mobile phase was as follows: solution A contained alkaline pH buffer and solution B contained acetonitrile. Chromatographic purity for linagliptin samples occurred before and after degradation were analyzed.

### 4.5. UPLC-MS Condition

The chromatographic conditions for the UPLC-MS study were the same as those for the UPLC-PDA method. The UPLC-MS analysis was performed using a UPLC instrument (Waters, Eschborn, Germany), connected with a single quadrupole detector (SQD) mass spectrometer and an electrospray ionization (ESI) source. The operating conditions for the MS scan of the linagliptin compound in positive electrospray ionization mode were optimized as follows: capillary voltage: 1.0 kV, source temperature: 120 °C, desolvation nitrogen flow: 800 L/Hr, desolvation temperature: 350 °C, cone nitrogen flow: 50 L/Hr, cone voltage: 30 V. The high-purity nitrogen was used as a nebulizing gas with a nebulizer pressure set to 60 psi and as drying gas with a flow rate of 12 L/min and the temperature set to 300 °C. The spectrometer was operated in the measurement range from 100 *m*/*z* to 1500 *m*/*z*. The instrument was controlled by Empower software (Waters, Eschborn, Germany; version no. 7.30.00.00).

The chromatographic separation was performed on UPLC BEH C18 (2.1 mm × 100 mm; 1.7 µm) column (Waters, Eschborn, Germany), using reverse phase gradient elution. The gradient method involves two mobile phases (A and B). The composite of these solutions in the mobile phase was as follows: solution A contained alkaline pH buffer and solution B contained acetonitrile. Using the above method, an analysis was performed to identify linagliptin impurities resulting from forced degradation.

### 4.6. High-Resolution Mass Spectrometry Analysis

A high-resolution mass spectrometry method for the confirmation of process-related impurities and degradation products of the intermediate product linagliptin was developed using an ultra-performance liquid chromatography system, Acquity I-Class BSM Plus (Waters, Eschborn, Germany), coupled with a photodiode array detector (UPLC-PDA), and a high-resolution mass spectrometer of the XEVO G2-XS Q-ToF (Waters, Eschborn, Germany). The chromatographic separation was performed on a UPLC BEH C18 (2.1 mm × 100 mm; 1.7 µm) column (Waters, Eschborn, Germany), using reverse phase gradient elution. The gradient method involves two mobile phases (A and B). The composite of these solutions in the mobile phase was as follows: solution A contained alkaline pH buffer and solution B contained acetonitrile. Using the above procedure, an analysis was performed to identify linagliptin impurities resulting from forced degradation. Electrospray ionization (ESI) was used as the ionization source. The MS scan conditions for the linagliptin substance in positive electrospray ionization were optimized as follows: capillary voltage: 0.8 kV, source temperature: 120 °C, desolvation gas flow: 800 L/h, desolvation temperature: 550 °C, cone gas flow: 50 L/h, cone voltage: 40 V. High-purity nitrogen was used as the nebulizing gas. The spectrometer operated in a scanning mode (100 ± 1200) *m*/*z*.

## 5. Conclusions

The conducted forced degradation studies have provided valuable insights into the behavior of linagliptin under various stress factors. Understanding the conditions leading to the formation of specific impurities is crucial for addressing stability-related concerns. By identifying the substance’s sensitivity to particular stressors, measures can be taken to minimize or prevent the generation of degradants during synthesis and establish suitable storage methods for enhanced stability.

In conclusion, the coupling of LC-PDA-MS with high-resolution mass spectrometry analysis successfully elucidated the degradation products and pathways of linagliptin. Linagliptin is commonly used in the treatment of type 2 diabetes mellitus, a condition characterized by elevated blood sugar levels due to the body’s impaired production or utilization of insulin. The observed vulnerability to acid hydrolysis and oxidative conditions underscores the necessity of comprehensive drug stability assessments. The identified degradants highlight the importance of avoiding acidic and oxidative environments during production, storage, and transportation. This study also employed the LC-MS to identify significant impurities resulting from acidic and oxidative degradation. In total, two acidic degradants, AD1 and AD2, were characterized, with AD1 arising from partial hydrolysis of the quinazoline ring and AD2 being a linagliptin dimer formed by acid-catalyzed aza-enolization. Additionally, four oxidation-induced degradants, OX 1 to OX 4, were identified. Notably, OX 1, a previously undescribed impurity, was proposed to result from the oxidation of tertiary nitrogen in the pyridinium ring. Efforts were made to identify isomeric impurities, OX 2 and OX 3, with *m*/*z* = 487.30069, and based on MS analysis and chemical knowledge, their structures were proposed as Z and E isomers of the oxime formed due to primary nitrogen oxidation. The compound OX 4, a known linagliptin derivative, where the primary amine is converted to its corresponding nitro compound, was also identified.

The identification of these degradation products is pivotal in drug manufacturing, providing insights into the potential impurities and their implications for drug safety. This research not only advances our understanding of linagliptin’s behavior but also contributes to the broader field of Active Pharmaceutical Ingredients stability studies.

## Figures and Tables

**Table 1:** List of identified degradation products of linagliptin (relative retention time (RRT)).

Compound	RRT *	Formula Weight	Molecular Formula	Chemical Structure	Source
Linagliptin	1.00	472.54	C_25_H_28_N_8_O_2_	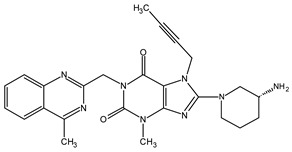	Target compound
AD 1	1.06	491.54	C_25_H_29_N_7_O_4_	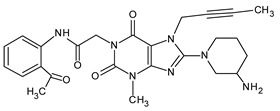	Acid degradation
AD 2	1.28	945.08	C_50_H_56_N_16_O_4_	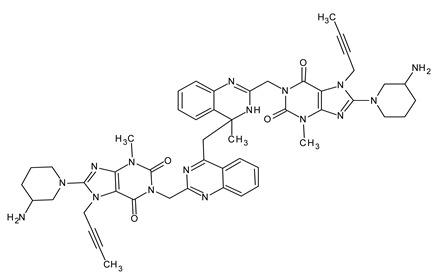	Acid degradation
OX 1	0.96	488.54	C_25_H_28_N_8_O_3_	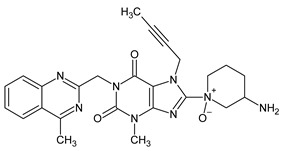	Oxidation
OX 2 OX 3	1.04 1.07	486.52	C_25_H_26_N_8_O_3_	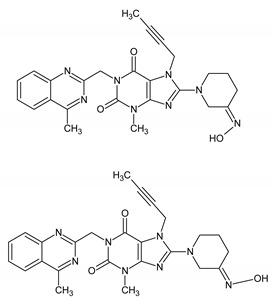	Oxidation
OX 4	1.31	502.52	C_25_H_28_N_8_O_4_	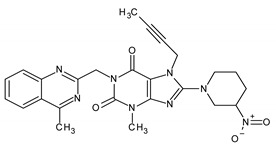	Oxidation

* The Relative Retention Time (RRT) is calculated by dividing the retention time (RT) of the degradant by the retention time of Linagliptin.

**Table 2:** Degradation condition of linagliptin and calculated degree of degradation.

Degradation Type	Experimental Conditions	Sampling Conditions	Degradation Time	Degree of Degradation
Acidic degradation	0.1 M HCl	60 °C	24 h	16.42%
Basic degradation	0.1 M NaOH	60 °C	10 days	2.56%
Oxidative degradation	3% H_2_O_2_	60 °C	24 h	35.86%
Thermal degradation	Heat chamber	60 °C	10 days	0.05%
UV-VIS photolysis	2.4 million lux hours 400 watt hours/square meter	60 °C 60% humidity	-	0.30%

## Data Availability

The authors confirm that the data supporting the findings of this study are available within the article and its Supplementary Material.

## Supplementary Materials

The following supporting information can be downloaded at: https://www.mdpi.com/article/10.3390/ijms25052591/s1.
