# Supplementary material for: Identification and Structural Characterization of Degradation Products of Linagliptin by Mass Spectrometry Techniques
Source: Int J Mol Sci. 2024 Feb 23;25(5):2591. doi: 10.3390/ijms25052591 (PMC10932084; doi:10.3390/ijms25052591)
Supplement: Supplementary file 1 [file ijms-25-02591-s001.zip › ijms-2865840-supplementary.pdf]

a

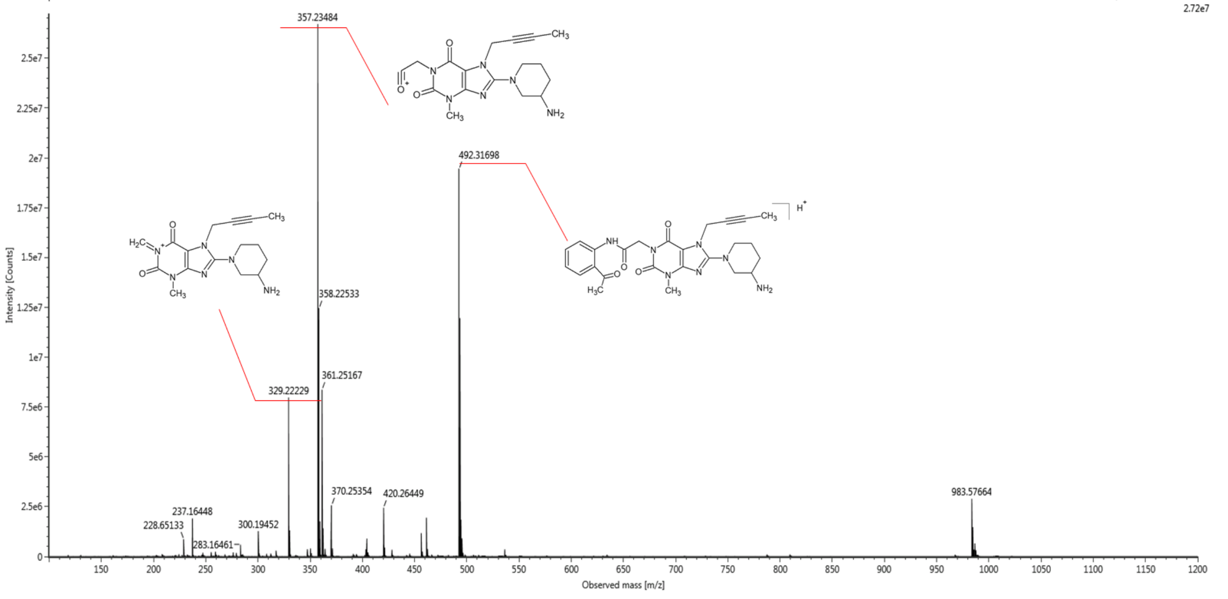

b

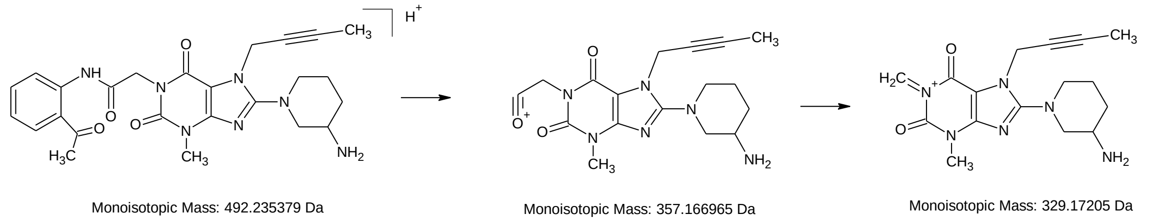

Figure S1. Electrospray positive ionization - mass spectrometry (ESI-MS) spectrum of AD1 recorded using the XEVO G2-XS Q-ToF high resolution mass spectromete (a) and pfragmentation pattern of AD1 (b)

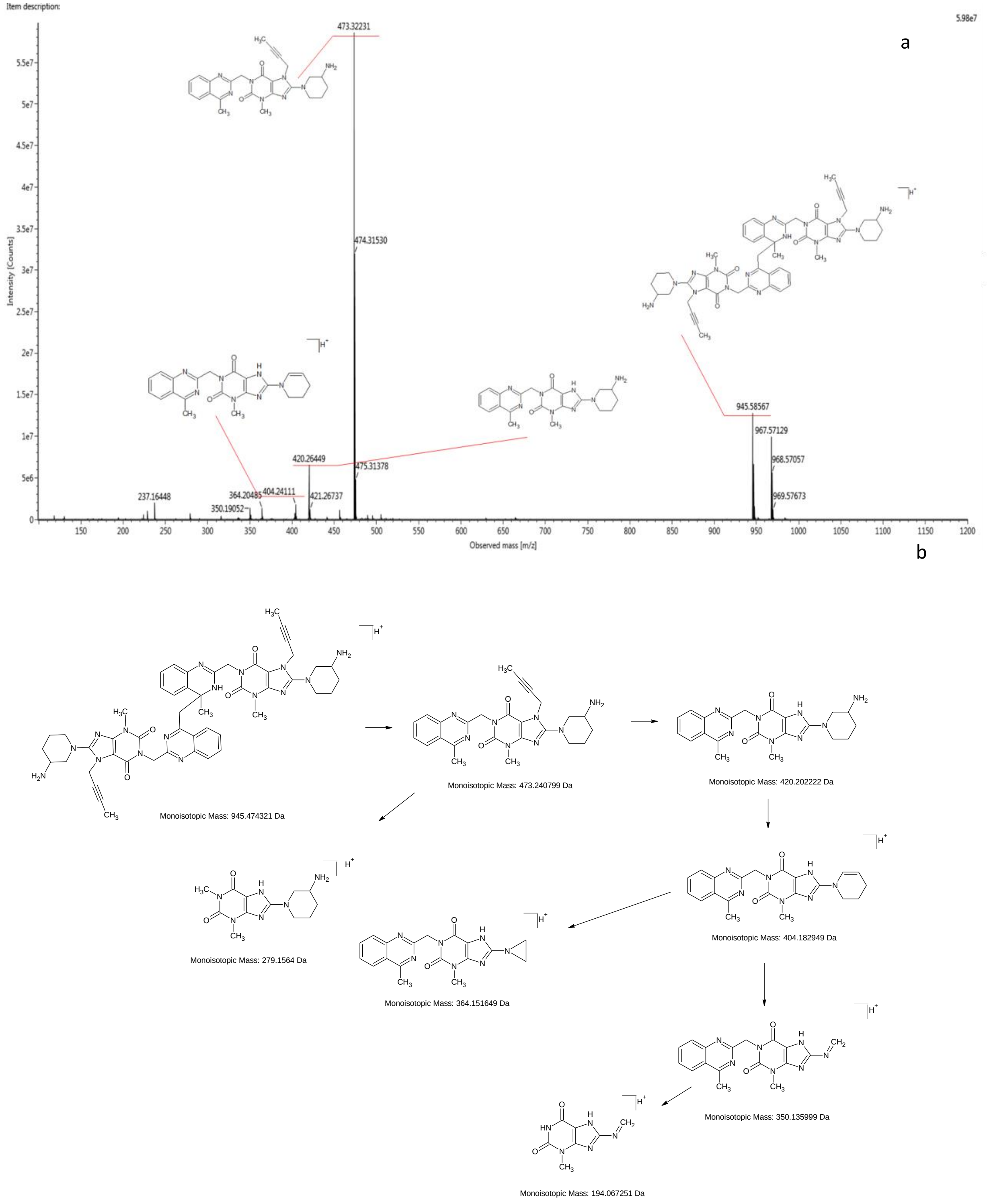

Figure S2. Electrospray positive ionization - mass spectrometry (ESI-MS) spectrum of AD2 recorded using the XEVO G2-XS Q-ToF high resolution mass spectromete (a) and proposed fragmentation pattern of AD2 (b)

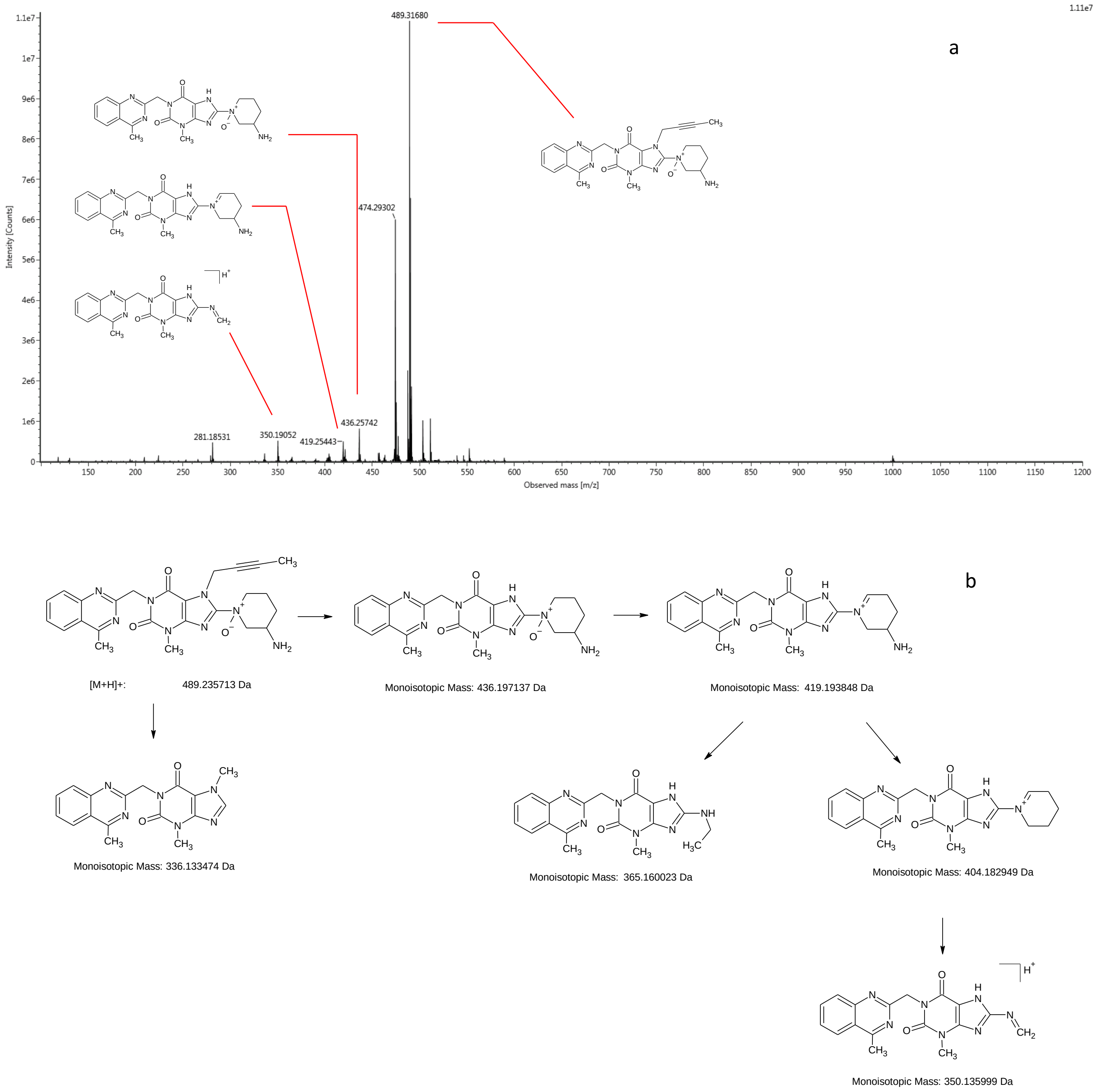

Figure S3. Electrospray positive ionization - mass spectrometry (ESI-MS) spectrum of OX1 recorded using the XEVO G2-XS Q-ToF high resolution mass spectromete (a) and proposed fragmentation pattern of OX1 (b)

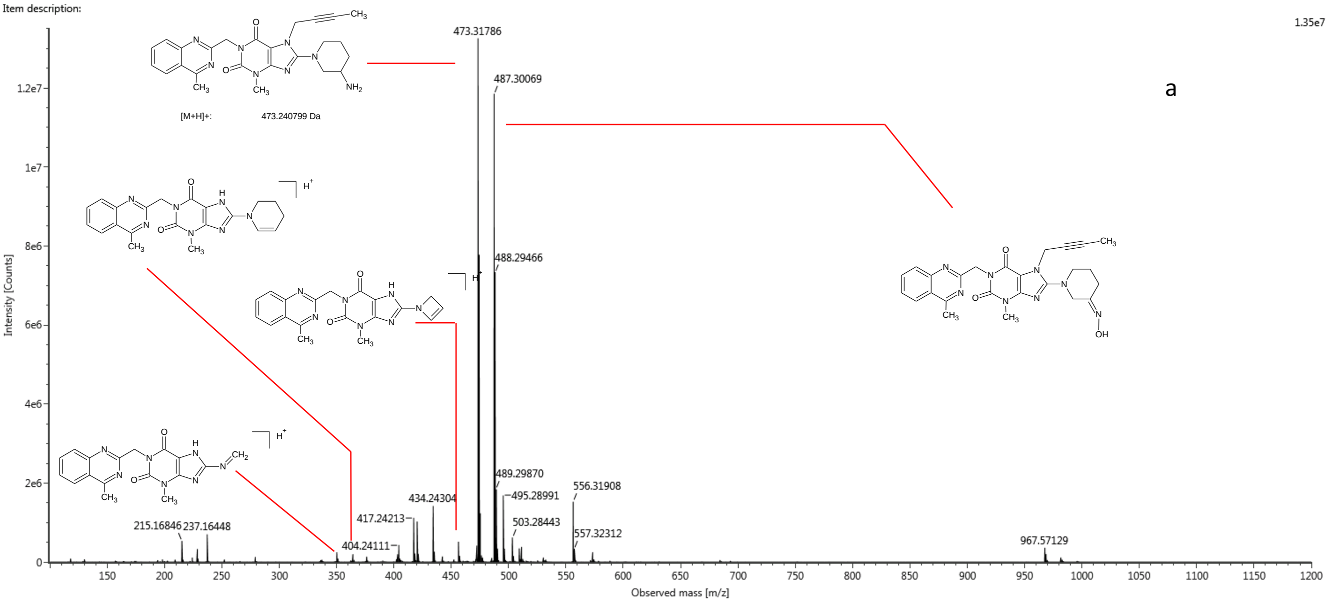

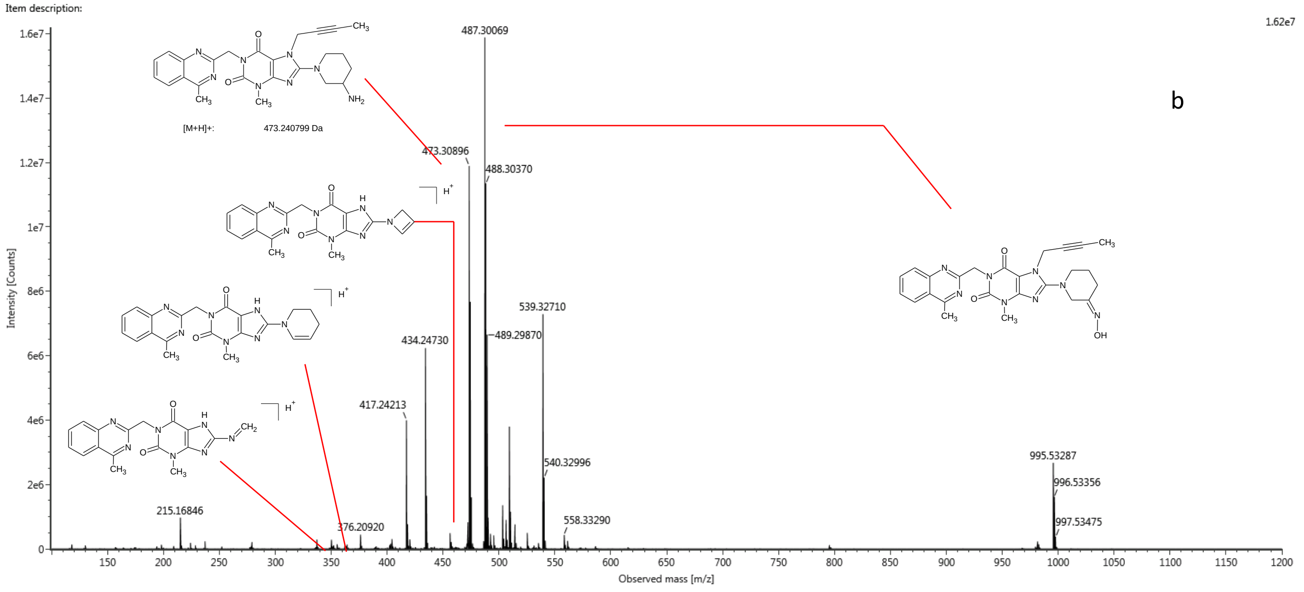

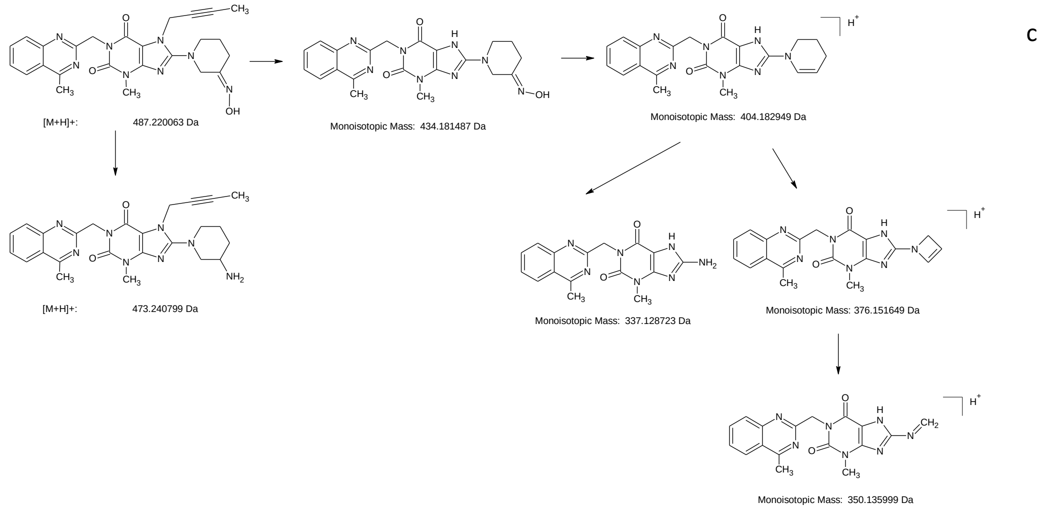

Figure S4. Electrospray positive ionization - mass spectrometry (ESI-MS) spectrum of OX2 (a) and OX3 (b) recorded using the XEVO G2-XS Q-ToF high resolution mass spectromete and proposed fragmentation pattern of OX2 and OX3 (c)

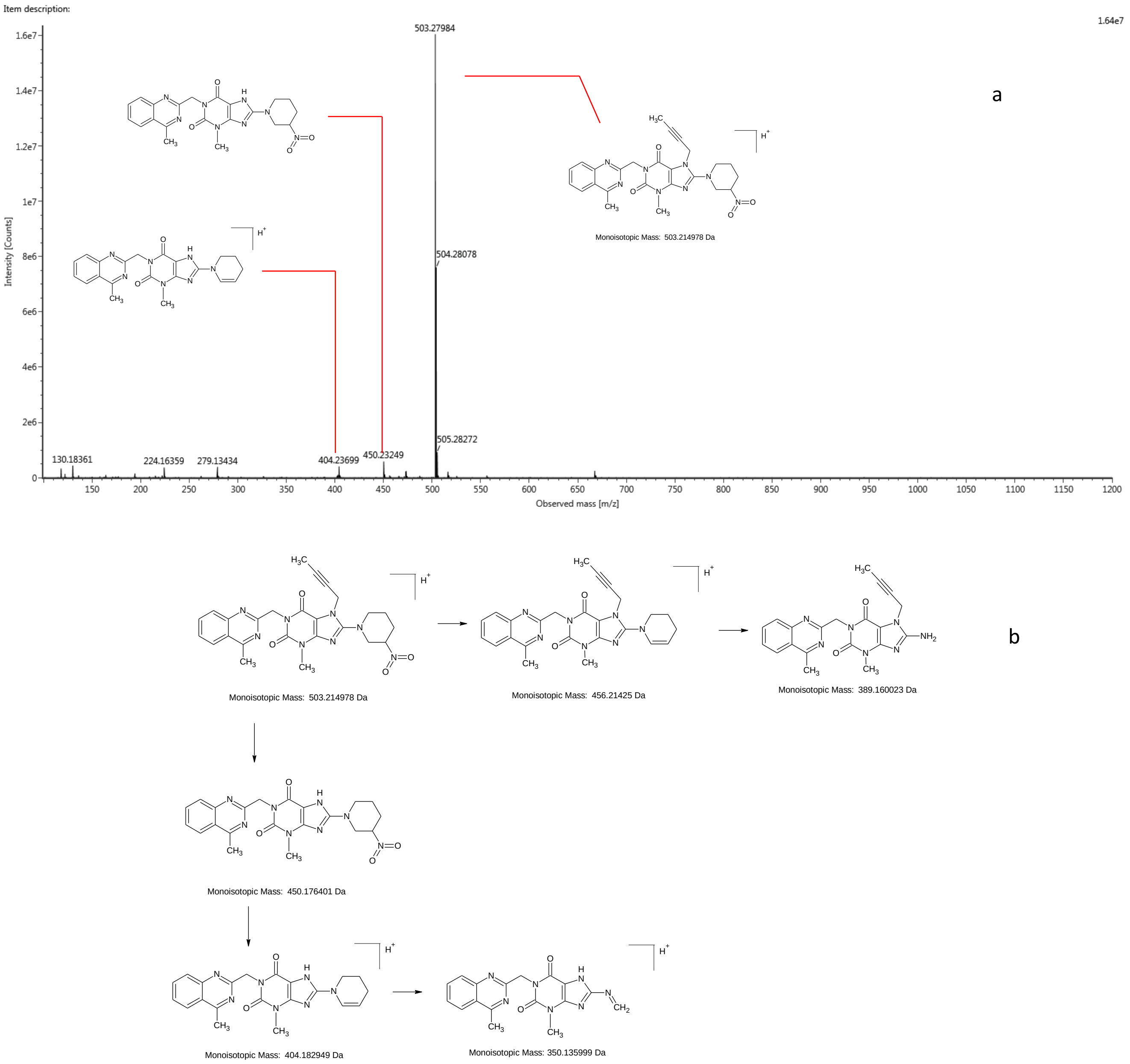

Figure S5. Electrospray positive ionization - mass spectrometry (ESI-MS) spectrum of OX4 recorded using the XEVO G2-XS Q-ToF high resolution mass spectromete (a) and proposed fragmentation pattern of OX4 (b)
